# Altered microbiota, fecal lactate, and fecal bile acids in dogs with gastrointestinal disease

**DOI:** 10.1371/journal.pone.0224454

**Published:** 2019-10-31

**Authors:** Amanda B. Blake, Blake C. Guard, Julia B. Honneffer, Jonathan A. Lidbury, Jörg M. Steiner, Jan S. Suchodolski

**Affiliations:** Gastrointestinal Laboratory, Texas A&M University, Texas, United States of America; University of Minnesota Twin Cities, UNITED STATES

## Abstract

The intestinal microbiota plays an important role in health and disease and produces, through fermentative reactions, several metabolic products, such as lactate, that can affect the host. The microbiota also interacts with and metabolizes compounds produced by the host, such as primary bile acids. Lactate and bile acids (BA) are of particular interest in gastrointestinal diseases because they have been associated with metabolic acidosis and bile acid diarrhea, respectively. The objectives of this study were to validate an enzymatic assay to quantify D-, L-, and total lactate in canine feces, and to characterize fecal lactate and BA concentrations as well as bacterial abundances in healthy dogs and dogs with gastrointestinal diseases. Fecal samples were collected from 34 healthy dogs, 15 dogs with chronic enteropathy (CE), and 36 dogs with exocrine pancreatic insufficiency (EPI). Lactate was quantified with an enzymatic assay, BA with gas chromatography-mass spectrometry, and 11 bacterial groups with qPCR. A fecal lactate reference interval was established from 34 healthy dogs and was 0.7–1.4 mM, 0.3–6.0 mM, and 1.0–7.0 mM for D-, L-, and total lactate, respectively. The assay to measure D-, L-, and total lactate in canine fecal samples was linear, accurate, precise, and reproducible. Significant increases in fecal lactate and decreases in secondary BA concentrations were observed in dogs with CE and dogs with EPI. Dogs with EPI had an increased abundance of *Escherichia coli*, *Lactobacillus*, and *Bifidobacterium*; a decreased abundance of *Fusobacterium* and *Clostridium hiranonis*; and a higher Dysbiosis Index when compared to healthy dogs. Further studies are necessary to determine the clinical utility of lactate and BA quantification in canine feces. These metabolites suggest functional alterations of intestinal dysbiosis and may become promising targets for further elucidating the role of the microbiota in health and disease.

## Introduction

There is mounting evidence defining the relationship between the intestinal microbiota, the metabolites it produces, and health and disease [1]. Recent molecular studies have revealed profound alterations in the intestinal microbial communities of dogs with various gastrointestinal (GI) diseases [2–5]. Methods to characterize the intestinal microbiota have evolved from traditional bacterial culture methods to high-throughput sequencing of the entire metagenome. However, quantitative polymerase chain reaction (qPCR) is often employed to measure abundance of specific bacterial groups.

The Canine Microbiota Dysbiosis Index (DI), a qPCR-based tool that is available commercially for diagnostics and disease monitoring, was recently developed to identify dysbiosis or changes in specific bacterial groups characteristic of dogs with chronic enteropathy (CE) [6, 7]. The DI quantitatively measures the abundance of total bacteria and seven bacterial groups (*Faecalibacterium* spp., *Turicibacter* spp., *Streptococcus* spp., *Escherichia coli*, *Blautia* spp., *Fusobacterium* spp., and *Clostridium hiranonis*) and is expressed as a single number where a value below zero is indicative of a normal microbiota. Additionally, using a threshold of zero, the DI algorithm had 74% sensitivity and 95% specificity to separate healthy dogs (n = 95) and dogs with CE (n = 106) [6]. Numerous studies using sequencing and/or qPCR-based methods have also shown these bacterial groups to be altered in dogs with GI disease [3, 8–12]. Recently, Minamoto et al. demonstrated that dysbiosis shown in 16S rRNA Illumina sequencing was accurately reflected by the DI in dogs with CE [8].

Additionally, PICRUSt (Phylogenetic Investigation of Communities by Reconstruction of Unobserved States) [13] or whole metagenome sequencing can be used to characterize changes in functional gene families that occur in diseased states. Furthermore, analysis of metabolic products present in the GI tract and feces through targeted and untargeted approaches can elucidate changes in functional and metabolic pathways. All of this information can aid in studying pathogenesis of diseases as well as in discovering novel biomarkers of disease or novel therapeutic targets.

The intestinal microbiota plays an essential role in maintaining host health by defending against pathogens, promoting development of a healthy immune system, and providing beneficial metabolites to host epithelial cells [14]. For example, through fermentative reactions, intestinal bacteria can convert complex carbohydrates into short chain fatty acids (SCFAs) that the host can then absorb and utilize [15]. In humans, it appears that certain metabolites produced by the intestinal microbiota may influence host metabolism [16]. Although the current literature characterizing intestinal microbiota and microbial metabolites in dogs is very descriptive, we have yet to make clear connections with functional and clinically relevant consequences. Furthermore, changes to the pool of microbial metabolites can occur without concurrent changes in the microbial populations [17]. This suggests that examining the microbiota and their metabolites together is crucial.

Recently, Minamoto et al. [12] examined the fecal microbiota and serum metabolite profiles in dogs with inflammatory bowel disease (IBD) and found that although several major bacterial groups were altered, only four named metabolites were significantly altered in serum (gluconolactone, hexuronic acid, ribose, and 3-hydroxybutanoic acid). Interestingly, studies in humans looking at a single microbial metabolite, D-lactate, showed that concentrations can become increased in feces while concentrations in the serum remain normal [18, 19]. Additionally, preliminary work using an untargeted metabolomics approach identified several hundred significantly altered metabolites in feces of dogs with CE, including lactate and secondary bile acids [20]. These studies all point to the need for examining metabolites in the feces in addition to serum.

Lactate is produced by the host cells [21, 22] and intestinal microbiota [23–25] in two isoforms: D-lactate and L-lactate. Another source of D- and L-lactate in the GI tract is dietary intake of fermented foods, such as yogurt and sauerkraut [26]. Lactate-producing bacteria are often used in these foods as well as probiotics because they can have beneficial properties in the gut, such as lowering luminal pH and countering pathogenic bacteria [27, 28]. However, when the systems of lactate production, absorbance, or clearance become altered, lactate can accumulate in the feces or serum.

Certain diseases or conditions associated with hypoperfusion, hypoxia, maldigestion, or metabolic disease can cause an abundance of lactate in the serum, leading to lactic acidosis. A thorough review of lactic acidosis is provided elsewhere [29, 30]. D-lactic acidosis, but not L-lactic acidosis, is characterized by neurological signs such as ataxia, confusion, blurred vision, lethargy, and irritability [30]. D-lactic acidosis has been reported in diseases associated with alterations in the intestinal microbiota, such as short bowel syndrome in humans [31], diarrhea in calves [32], and exocrine pancreatic insufficiency (EPI) in a cat [33]. In part because the different lactate isoforms produce distinct clinically relevant types of acidosis and are associated with different bacterial groups, it is of interest to measure D- and L-lactate in feces. However, conventional approaches with gas chromatography-mass spectrometry (GC-MS) or high-pressure liquid chromatography (HPLC) require complex sample preparation and expensive laboratory equipment that is often available only to reference laboratories. A cost-effective and relatively quick method for measuring both isoforms of lactate in feces is enzymatic spectrophotometry assays. Many studies have utilized these assays previously in human feces [18, 34], cow feces [35, 36], and murine feces [37], making them a promising assay for the measurement of lactate in canine feces.

Bile acids are another promising target for analysis in feces because of their important role in host metabolism and their direct relationship with the intestinal microbiota. Briefly, primary bile acids (cholic acid and chenodeoxycholic acid) are synthesized in the liver from cholesterol and then conjugated with the amino acids glycine and taurine [38] before being released into the intestinal lumen. There, they interact with fat to form micellar structures and facilitate absorption of fat into enterocytes, resulting in about 95% of the bile acids being reabsorbed as well [39]. The intestinal microbiota affects bile acid metabolism through deconjugation and dehydroxylation of primary bile acids to form secondary bile acids, such as lithocholic acid, deoxycholic acid, and ursodeoxycholic acid [40]. Secondary bile acids have been associated with immune regulation [41, 42] and, conversely, carcinogenesis [43, 44]. This may suggest that bile acids are essential to maintaining homeostasis in the GI tract but that an overabundance of secondary bile acids could be toxic.

With lactate and secondary bile acids being major metabolites of bacterial origin, examining concentrations in the feces should aid in detecting any disturbances to the GI microbiota. Therefore, the objectives of this study were to validate an enzymatic assay for the quantification of D-, L-, and total lactate in canine feces, and to characterize fecal lactate and bile acid concentrations as well as bacterial abundances in healthy dogs and dogs with gastrointestinal diseases.

## Materials and methods

### Ethics statement

All dogs included in our analysis were client owned, and all feces collected were naturally voided. The owners consented to the research and no personal details of the owners were collected. An approval by an ethics committee at Texas A&M University is only required if the study may be associated with possible pain or suffering of the patients due to study procedures. As this study was not involving any animals directly, but exclusively through using historical information and gathering of feces after defecation, such an approval by the Institutional Animal Care and Use Committee was not necessary and thus not sought.

### Animals and sample collection

All dogs were at least 1 year of age, were housed at the owners’ premises, and had not received antibiotics for at least 3 weeks prior to sample collection. Table 1 summarizes the metadata for dogs included in the study, and Table 2 summarizes fecal collection and shipping conditions of samples. Fecal samples from 12 dogs with treated EPI were collected prospectively from owners around the United States, after being recruited through an EPI forum. All other fecal samples were analyzed retrospectively having been initially collected for previous studies. Client consent was obtained before enrollment of dogs.

**Table 1:** Metadata for dogs included in the study, if available.

	Healthy	CE	Untreated EPI	Treated EPI
**Age (years; median, range)**	4, 1–12	6, 3–11	2, 1.1–4	3.5, 1–14
**Gender (female/male)**	20/14	2/4	2/5	21/7
**Breed (n)**	Mixed Breed (14), GSD (7), Miniature Schnauzer (3), Mastiff (2), Bull Terrier (1), Chinese Crested Dog (1), Dachshund (1), English Cocker Spaniel (1), Husky (1), Miniature Poodle (1), Vizsla (1), Weimaraner (1)	Mixed Breed (3), Austrlian Shepherd (1), Bernese Mountain Dog (1), Bolognese (1), Border Collie (1), Border Terrier (1), Golden Retriever (1), GSD (1), Jack Russell Terrier (1), Labrador Retriever (1), Miniature Pinscher (1), West Highland White Terrier (1)	GSD (4), Bichon Frise (1), Chihuahua (1), Corgi (1)	GSD (17), Mixed Breed (2), Chihuahua (1), Corgi (1), Husky (1), Labrdoodle (1), Pit bull (1), Rottweiler (1), Rough Collie (1), Spanish Water Dog (1), Springer Spaniel (1), Yorkshire Terrier (1)
**Diet (n)**	Maintenance diet (27)	Maintenance diet (11), Maintenance+ homemade (3), Homemade only (1)	Maintenance diet (5), Maintenance+ homemade (1)	Maintenance diet (23), Maintenance+ homemade (4)

CE = chronic enteropathy, Untreated EPI = dogs with exocrine pancreatic insufficiency not receiving enzyme replacement therapy, Treated EPI = dogs with exocrine pancreatic insufficiency receiving enzyme replacement therapy, GSD = German Shepherd dog

**Table 2:** Fecal sample collection details.

Group and Number	Collection	Storage and Shipping Conditions
healthy (n = 34)	single TP (n = 16), 3 consecutive bowel movements, pooled (n = 18)	-80⁰C, ice packs overnight shipping
CE (n = 15)	single TP	-80⁰C, dry ice and ice packs overnight shipping
treated EPI (n = 29)	single TP (n = 12), 3 consecutive bowel movements, pooled (n = 17)	-80⁰C, ice packs, overnight shipping
untreated EPI (n = 7)	3 consecutive bowel movements, pooled	-80⁰C, ice packs, overnight shipping

TP = time point, CE = chronic enteropathy, treated EPI = dogs with exocrine pancreatic insufficiency receiving enzyme replacement therapy, untreated EPI = dogs with exocrine pancreatic insufficiency not receiving enzyme replacement therapy

Healthy control dogs (n = 34) had no clinical signs of GI disease. Fecal lactate concentrations from all healthy dogs were used to calculate a reference interval. However, due to the limited amount of feces collected, bile acid concentrations and lactic acid bacterial abundances were quantified in a subset of healthy dogs (n = 17 and n = 18, respectively).

Dogs with chronic enteropathy (CE; n = 15) had GI signs for more than 3 weeks and had common causes for intestinal disease (e.g. parasites, enteropathogens) excluded. Definitive diagnosis was reached after endoscopy and histopathologic examination of intestinal biopsies, with inflammatory findings consistent with CE. Feces were collected prior to bowel cleanse and endoscopy.

Dogs with exocrine pancreatic insufficiency (EPI; n = 36) were diagnosed by a serum trypsin-like immunoreactivity (cTLI) of less than 2.5 μg/L. Dogs were separated into two groups: those that were receiving pancreatic enzyme replacement therapy at the time of fecal collection (treated EPI, n = 29) and those that had not yet received therapy (untreated EPI, n = 7). Pancreatic enzyme replacement therapy is the standard treatment for dogs with EPI, and consists of mixing commercially available pancreatic enzyme supplements with meals. Information on duration of enzyme replacement therapy was obtained when available (treated EPI; n = 12). Due to the limited amount of feces collected, fecal lactate and bile acid data could not be obtained for 2 of the dogs with untreated EPI.

### Quantification of fecal lactate

Concentrations of D-, L-, and total lactate were measured in fecal samples by utilizing the protocol of Rul et al. [37] with modifications (http://dx.doi.org/10.17504/protocols.io.hrqb55w. [PROTOCOL DOI]). Briefly, 120–130 mg fecal aliquots were diluted in 750 μL of 0.1 M triethanolamine buffer (pH 9.15). Samples were vortexed and centrifuged at 13,000 x g for 5 minutes at 4°C. Next, 495 μL of the supernatant was deproteinized with 10 μL of 6 M trichloroacetic acid, vortexed, and placed in an ice bath for 20 minutes. Then samples were centrifuged at 4,500 x g for 20 minutes at 4°C, and 400 μL of supernatant was diluted with 1600 μL 0.1 M triethanolamine buffer (pH 9.15) to achieve a neutral or alkaline pH (between 7 and 10). These deproteinized fecal extracts were used for spectrophotometric analysis using a commercially available enzymatic kit (D-/L-Lactate Enzymatic Kit, R-Biopharm Inc., Darmstadt, Germany) with modifications to the manufacturer protocol for use with a 96-well plate format. A detailed protocol for the quantification of fecal lactate is provided in S1 Protocol.

### Analytical validation of the assay to measure fecal lactate

Surplus homogenized canine fecal samples from seven dogs were used for analytical validation. Validation variables tested were lower limit of detection, lower limit of quantification, dilutional parallelism, spiking recovery, and intra- and inter-assay variability. Deproteinized fecal extract stability was evaluated by measuring D- and L-lactate in seven sample extracts at baseline, after 24 hours of storage at 4°C, and after 28 days of storage at -80°C. The reference intervals for canine fecal lactate concentrations were calculated from the central 95^th^ percentile of 34 healthy dogs. These dogs were determined to be healthy based on responses from owner questionnaires. The full validation protocol is available in S2 Protocol.

### Quantification of bacterial groups in feces

A 600–1,200 mg aliquot of feces was lyophilized for each sample and dry matter weights were obtained. DNA was extracted from the lyophilized fecal samples with a commercially available kit (PowerSoil® DNA Isolation Kit, MOBIO Laboratories, Inc., Carlsbad, CA, USA) and the DNA concentration measured (Spectrophotometer, Nanodrop 1000, Thermo Scientific, Rockford, IL, USA). DNA was normalized for concentration on a 96-well plate using a pipetting robot (epMotion 5075 Vacuum TMX, Eppendorf AG, Hamburg, Germany). Separate real-time qPCR assays were used to amplify and quantify DNA from total bacteria and 7 bacterial groups known to be relevant in GI diseases (*Faecalibacterium* spp., *Turicibacter* spp., *Streptococcus* spp., *Escherichia coli*, *Blautia* spp., *Fusobacterium* spp., and *Clostridium hiranonis*) using protocols and primers described in S1 Table.

Separate real-time qPCR assays were also used to quantify abundance of several lactate-producing bacterial groups (*Lactobacillus* spp., *Bifidobacterium* spp., and *Enterococcus* spp.) using protocols and primers described in S1 Table. These bacterial groups are relatively abundant members of the GI microbiota and therefore could influence luminal lactate production and subsequently affect fecal lactate concentrations.

SYBR-based reaction mixtures (total 10 μl) contained 5 μl SsoFast^™^ EvaGreen® Supermix (Bio-Rad Laboratories, CA, USA), 2.2 μl water, 0.4 μl of each primer (final concentration: 400 nM), and 2 μl of normalized DNA (final concentration: 5 ng/μl). A melt curve analysis was performed after the amplification cycles as follows: increments of 0.5°C from 65°C to 95°C for 5 seconds each. Samples were analyzed in duplicate, and a commercially available qPCR thermal cycler (CFX96TM, Bio-Rad Laboratories, CA, USA) was used for all qPCR assays. Data was expressed as log DNA (fg) of target bacterial group per 10 ng starting quantity total DNA, or LogSQ.

The abundance of bacterial DNA for each bacterial group was compared between diseased groups and healthy controls. Additionally, microbiota data were expressed as a previously described Dysbiosis Index [6, 7], where a Dysbiosis Index below zero is indicative of a normal microbiota.

### Quantification of fecal bile acids

The protocol for identification and quantitation of bile acids [10] was adapted and modified from methods previously described [45, 46]. An aliquot of 10–15 mg lyophilized feces was resuspended in 200 μL 1-butanol (HPLC grade, Sigma-Aldrich, St. Louis, MO) containing internal standards cholic acid-d_4_ and lithocholic acid-d_4_ (CDN Isotopes, Quebec, Canada). Next, 20 μL 37% HCl (American Chemical Society reagent, Sigma-Aldrich, St. Louis, MO) was added and the samples capped, vortexed, and incubated for 4 hours at 65°C. Then, samples were evaporated under nitrogen gas for 25 minutes at 65°C, or until dry, followed by the addition of 200 μL Sylon TMS derivatization agent (Supelco’s® Sylon HTP, HMDS + TCMS + Pyridine, 3:1:9 Kit, Sigma-Aldrich, St. Louis, MO, USA) and incubation for 30 minutes at 65°C. Samples were again evaporated under nitrogen gas for 25 minutes at 65°C, or until dry, and then resuspended in 200 μL hexane (HPLC grade, Sigma-Aldrich, St. Louis, MO), vortexed, and centrifuged for 10 minutes at 3,000 rcf. Then an aliquot of supernatant was transferred to a GC-MS vial insert for further downstream analysis.

Gas chromatography (GC) and mass spectrometry (MS) were carried out in-house on an Agilent model 6890N and 5975 inert Mass Selective Detector, respectively (Agilent Technologies, Santa Clara, CA, USA). The instrument was equipped with a 7683 Series autosampler and a capillary DB-1ms Ultra Inert column purchased from Agilent. Selected ion monitoring was used with a 20:1 split ratio after a 1 μL sample injection and inlet temperature of 250°C. Following injection, oven temperature was kept at 150°C for 1 minute, ramped at 21°C per minute to a final temperature of 276°C, then held at that temperature for 21 minutes. At 28 minutes, the oven was ramped to 325°C for 3 minutes for post run column cleaning. Helium was used as the carrier gas at an approximate flow rate of 1 mL/min, which varied slightly with pressure to account for retention time locking cholestane-d_4_ to elute at 11.4 minutes. Mass spectral data were analyzed using Agilent’s Enhanced Data Analysis in MSD ChemStation version D.02.002.275.

### Statistical analysis

All statistical analysis was performed with GraphPad Prism 8.0.2 (GraphPad Software, La Jolla, California, USA) or JMP® Pro 14.0.0 (64-bit, SAS Institute Inc.). A Shapiro-Wilk test for normality was used to determine the distribution of data (non-parametric). Kruskal-Wallis tests were used to evaluate differences in variables between diseased and healthy groups. For post hoc analysis, the Steel-Dwass test for multiple comparisons with control (healthy group) was used to independently compare ranks of each group to healthy dogs. Spearman’s rank correlation analysis was performed on the subset of dogs with EPI for which duration of enzyme therapy was known (n = 12) to determine correlations between duration of enzyme therapy and any measured variables. Fisher’s exact test was used to compare proportions of dogs with Dysbiosis Indexes above and below zero.

## Results

### Analytical validation of assay for measurement of fecal lactate

The lower limits of detection for D- and L-lactate concentrations were 0.0006 and 0.0002 g/L, respectively. The lower limits of quantification for D- and L-lactate concentrations were 0.0021 and 0.0008 g/L, respectively. Observed-to-expected ratios for dilutional parallelism ranged from 92% to 111% for D-lactate, 89% to 109% for L-lactate, and 88% to 104% for total lactate (S2 Table). Recovery for spiking sample extracts ranged from 96% to 103% for D-lactate, 96% to 119% for L-lactate, and 98% to 113% for total lactate (S3 Table). Average intra-assay coefficients of variation for D-, L-, and total lactate were 5%, 5%, and 4%, respectively (S4 Table). Average inter-assay %CVs for D-, L-, and total lactate were 24%, 20%, and 19%, respectively (S5 Table). Fecal lactate was stable in deproteinized fecal extracts for 24 hours of storage at 4°C (average %CV: 9, 4, 4, for D-, L-, and total lactate, respectively; S6 Table) and 28 days of storage at -80°C (average %CV: 4, 4, 3, for D-, L-, and total lactate, respectively; S7 Table). Canine fecal lactate reference interval was 0.7–1.4 mM, 0.3–6.0 mM, and 1.0–7.0 mM for D-, L-, and total lactate, respectively.

### Dogs with CE v healthy control dogs

**Fig 1:**
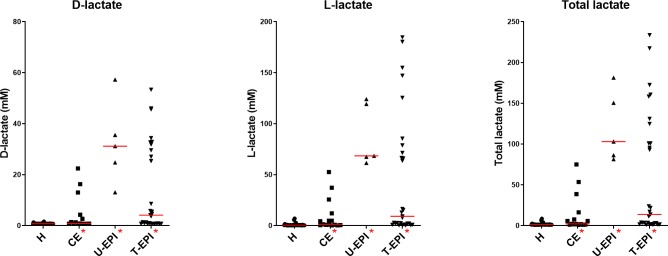
Lactate concentrations (mM) in the feces of healthy and diseased dogs. The groups on the x-axis are defined as follows: H = healthy dogs, AHD = dogs with acute hemorrhagic diarrhea, CE = dogs with chronic enteropathy, U-EPI = dogs with exocrine pancreatic insufficiency not receiving enzyme replacement therapy, T-EPI = dogs with exocrine pancreatic insufficiency receiving enzyme replacement therapy. Groups marked with a red asterisk are significantly different from the healthy control group.

**Fig 2:**
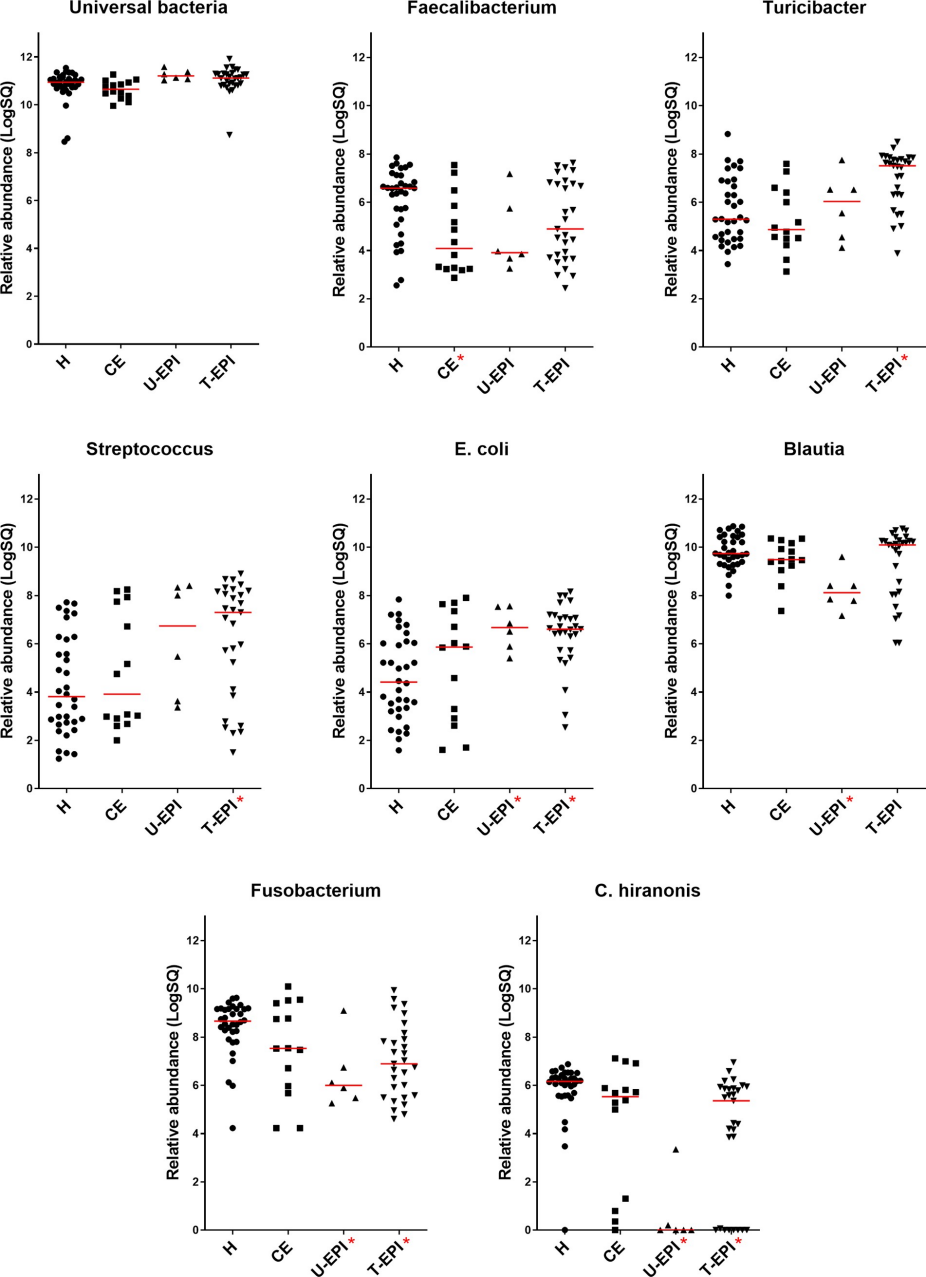
Bacterial abundances of those bacterial groups used to calculate Dysbiosis Index in the feces of healthy and diseased dogs. Abbreviations as in Fig 1.

**Fig 3:**
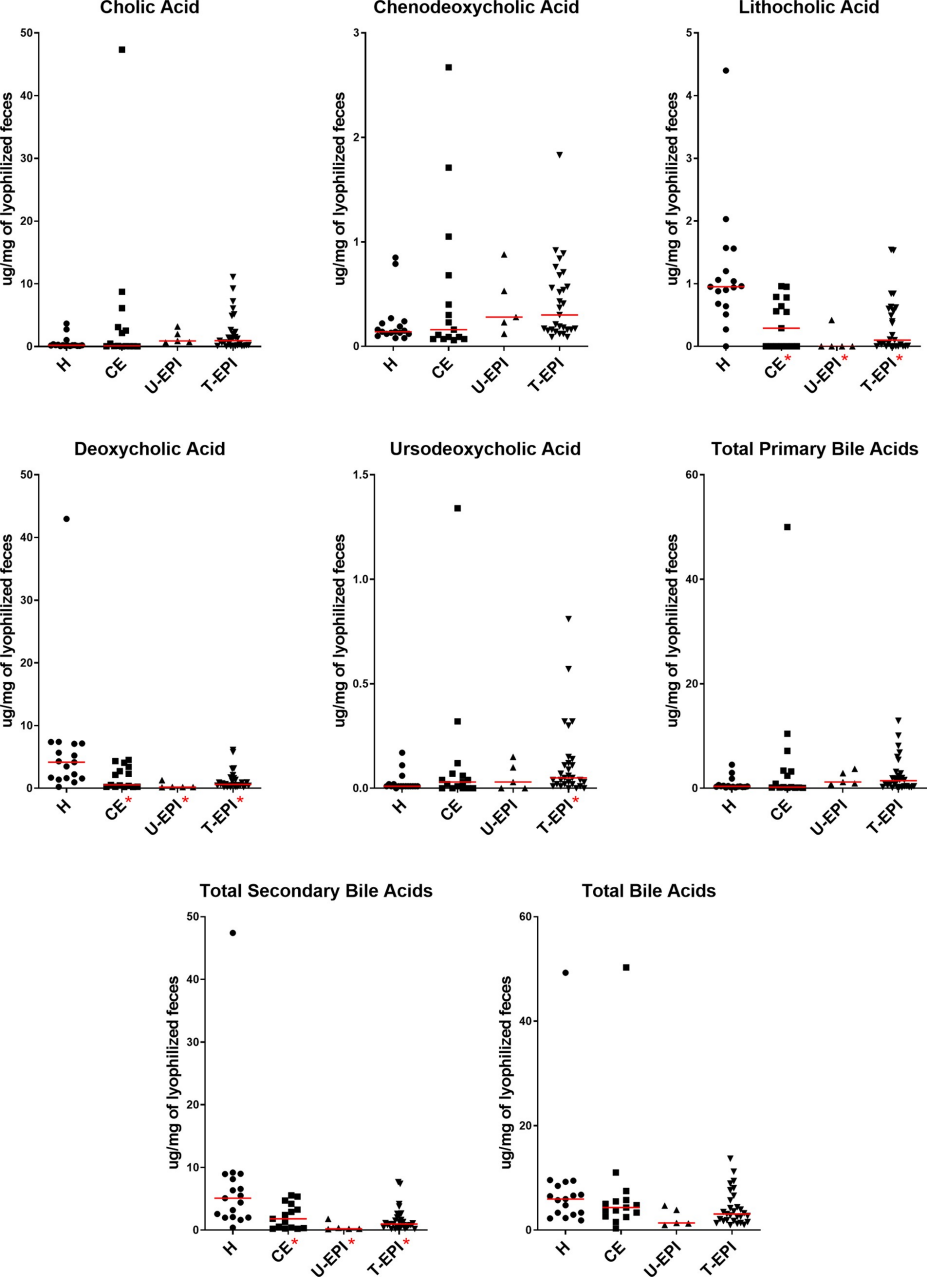
Bile acid concentrations (μg/mg of lyophilized feces) in the feces of healthy and diseased dogs. Abbreviations as in Fig 1.

D-, L-, and total lactate concentrations in feces were significantly higher in dogs with CE than in healthy control dogs (Table 3, Fig 1). One sample had poor quality DNA and therefore was removed from analysis of qPCR and DI. Abundances of *Faecalibacterium* were significantly lower in dogs with CE than in healthy control dogs (Table 3, Fig 2). Healthy dogs were more likely to have a DI less than zero when compared to dogs with CE (82% versus 36% respectively, p = 0.0044, Fisher’s exact test). Dogs with CE also had lower concentrations of the secondary bile acids lithocholic acid and deoxycholic acid, as well as lower concentrations of total secondary bile acids when compared to healthy control dogs (Table 4, Fig 3).

**Table 3:** D-, L-, and total fecal lactate concentrations (median [min-max] mM), abundance of bacterial groups (median [min-max] LogSQ), and Dysbiosis Index (median [min-max]) in diseased dogs compared to healthy control dogs with Steel-Dwass test.

	Healthy	CE	Untreated EPI	Treated EPI
**D-lactate**	0.8 [0.7–1.6]	0.9 [0.7–22.5];	31.2 [13.1–57.3];	4.1 [0.7–53.3];
		**p = 0.0099**	**p = 0.0017**	**p<0.0001**
**L-lactate**	0.5 [0.3–7.0]	1.4 [0.3–52.6];	68.5 [61.6–124.2];	9.3 [0.3–184.7];
		**p = 0.0176**	**p = 0.0019**	**p<0.0001**
**Total lactate**	1.3 [1.0–8.6]	2.2 [1.1–75.0];	103.1 [81.5–181.4];	13.9 [1.0–233.6];
		**p = 0.0130**	**p = 0.0019**	**p<0.0001**
**Universal**	10.94 [8.46–11.54]	10.65 [9.95–11.26];	11.21 [11.02–11.58];	11.12 [8.74–11.92];
		p = 0.0972	p = 0.0690	p = 0.1998
**Faecalibacterium**	6.58 [2.56–7.86]	4.09 [2.87–7.54];	3.91 [3.26–7.17];	4.90 [2.44–7.63];
		**p = 0.0303**	p = 0.1159	p = 0.1894
**Turicibacter**	5.30 [3.44–8.83]	4.87 [3.13–7.59];	6.03 [4.12–7.75];	7.51 [3.88–8.50];
		p = 0.7629	p = 0.9874	**p = 0.0006**
**Streptococcus**	3.81 [1.24–7.72]	3.91 [2.00–8.25];	6.74 [3.37–8.41];	7.31 [1.50–8.91];
		p = 0.7339	p = 0.1363	**p = 0.0060**
**Escherichia coli**	4.41 [1.59–7.84]	5.87 [1.61–7.91];	6.68 [5.40–7.56];	6.61 [2.54–8.17];
		p = 0.8044	**p = 0.0358**	**p = 0.0007**
**Blautia**	9.74 [8.00–10.87]	9.49 [7.36–10.36];	8.12 [7.16–9.60];	10.10 [6.04–10.77];
		p = 0.4958	**p = 0.0043**	p = 0.9681
**Fusobacterium**	8.66 [4.23–9.63]	7.53 [4.23–10.10];	6.00 [5.26–9.10];	6.89 [4.61–9.95];
		p = 0.5802	**p = 0.0240**	**p = 0.0021**
**Clostridium hiranonis**	6.16 [0.01–6.88]	5.53 [0.01–7.12];	0.01 [0.01–3.35];	5.36 [0.01–6.95];
		p = 0.1856	**p = 0.0012**	**p = 0.0009**
**Dysbiosis Index**	-3.2 [-7.2 to 4.3]	0.6 [-6.6 to 7.9];	6.5 [2.5 to 8.5];	0.8 [-6.8 to 8.5];
		p = 0.0638	**p = 0.0009**	**p = 0.0002**
**Lactobacillus**	3.27 [2.13–8.53]	3.94 [3.10–6.35];	7.17 [4.41–8.76];	6.68 [3.59–8.77];
		p = 0.2041	**p = 0.0247**	**p = 0.0002**
**Bifidobacterium**	3.49 [1.26–7.09]	3.02 [1.48–5.93];	6.82 [6.00–7.97];	6.42 [2.47–8.34];
		p = 0.9855	**p = 0.0046**	**p<0.0001**
**Enterococcus**	2.72 [0.01–5.77]	4.86 [1.61–6.39];	3.69 [1.62–7.40];	4.02 [0.87–6.21];
		p = 0.1201	p = 0.8357	**p = 0.0384**

**Table 4:** Bile acid concentrations (median [min-max] μg/mg lyophilized feces) and bile acid proportions (median [min-max] % of total bile acids) in diseased dogs compared to healthy control dogs with Steel-Dwass test.

	Healthy	CE	Untreated EPI	Treated EPI
**Cholic Acid**	0.21 [0.07–3.66]	0.10 [0.00–47.34];	0.90 [0.64–3.19];	0.93 [0.04–11.09];
		p = 0.9399	p = 0.0508	p = 0.1209
**Chenodeoxycholic Acid**	0.14 [0.08–0.85]	0.16 [0.06–2.67];	0.28 [0.12–0.88];	0.30 [0.09–1.83];
		p = 1.0000	p = 0.2433	p = 0.0533
**Lithocholic Acid**	0.95 [0.00–4.40]	0.29 [0.00–0.96];	0.00 [0.00–0.42];	0.10 [0.00–1.54];
		**p = 0.0040**	**p = 0.0079**	**p = 0.0002**
**Deoxycholic Acid**	4.16 [0.23–42.97]	0.54 [0.20–4.54];	0.19 [0.17–1.28];	0.62 [0.18–6.12];
		**p = 0.0317**	**p = 0.0050**	**p = 0.0003**
**Ursodeoxycholic Acid**	0.01 [0.00–0.17]	0.03 [0.00–1.34];	0.03 [0.00–0.15];	0.05 [0.00–0.81];
		p = 0.8855	p = 0.9899	**p = 0.0163**
**Total Primary Bile Acid**	0.37 [0.16–4.52]	0.22 [0.09–50.01];	1.19 [0.76–3.72];	1.44 [0.13–12.92];
		p = 0.9760	p = 0.0510	p = 0.0774
**Total Secondary Bile Acid**	5.11 [0.40–47.42]	1.81 [0.22–5.55];	0.21 [0.17–1.80];	0.98 [0.20–7.67];
		**p = 0.0230**	**p = 0.0050**	**p = 0.0003**
**Total Bile Acid**	5.95 [1.88–49.27]	4.33 [0.34–50.28];	1.39 [0.97–4.68];	3.10 [0.99–13.69];
		p = 0.7168	p = 0.0512	p = 0.0887
**% Secondary Bile Acid**	93.05 [11.79–96.92]	77.28 [0.53–98.20];	22.03 [4.38–38.49];	41.48 [4.11–95.60];
		p = 0.6254	**p = 0.0107**	**p = 0.0003**
**% Primary Bile Acid**	6.95 [3.08–88.21]	22.72 [1.80–99.47];	77.97 [61.51–95.62];	58.52 [4.40–95.89];
		p = 0.6254	**p = 0.0107**	**p = 0.0003**
**% Cholic Acid**	3.24 [1.56–81.76]	12.18 [0.00–94.15];	64.96 [42.63–82.00];	38.78 [1.58–85.86];
		p = 0.8941	**p = 0.0136**	**p = 0.0023**
**% Chenodeoxycholic Acid**	2.90 [1.38–13.13]	7.90 [1.54–20.03];	17.82 [12.34–20.38];	10.03 [2.14–26.32];
		p = 0.1702	**p = 0.0038**	**p<0.0001**
**% Lithocholic Acid**	16.93 [0.00–36.09]	11.45 [0.00–50.42];	0.00 [0.00–9.03];	2.42 [0.00–35.06];
		**p = 0.0476**	**p = 0.0118**	**p = 0.0458**
**% Deoxycholic Acid**	72.43 [6.82–87.21]	61.39 [0.49–84.76];	15.07 [4.27–27.29];	34.16 [3.20–78.62];
		p = 0.5463	**p = 0.0107**	**p = 0.0011**
**% Ursodeoxycholic Acid**	0.22 [0.09–4.97]	0.94 [0.00–26.61];	2.17 [0.00–11.55];	1.24 [0.00–21.30];
		p = 0.9521	p = 0.9263	**p = 0.0015**

### Dogs with EPI v healthy control dogs

**Fig 4:**
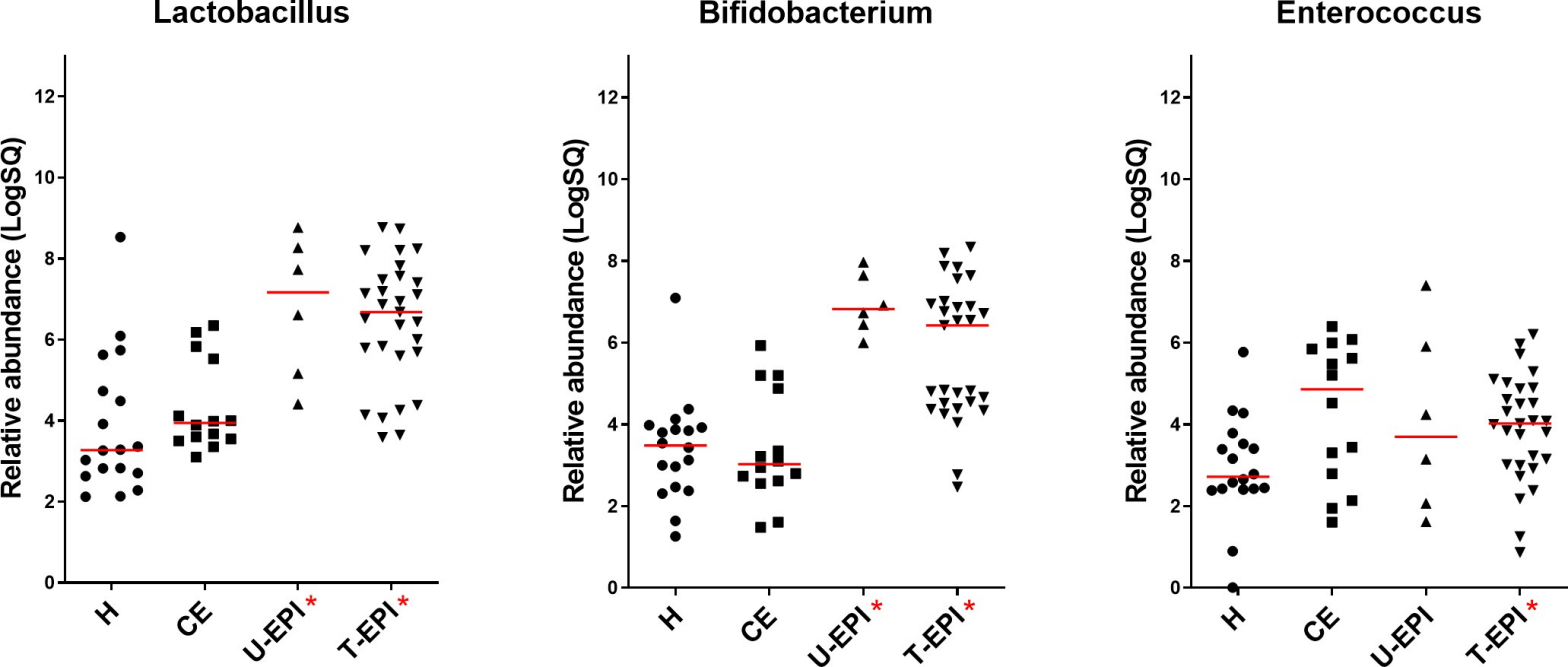
Abundance of specific lactate-producing bacterial groups in healthy and diseased dogs. Abbreviations as in Fig 1.

**Fig 5:**
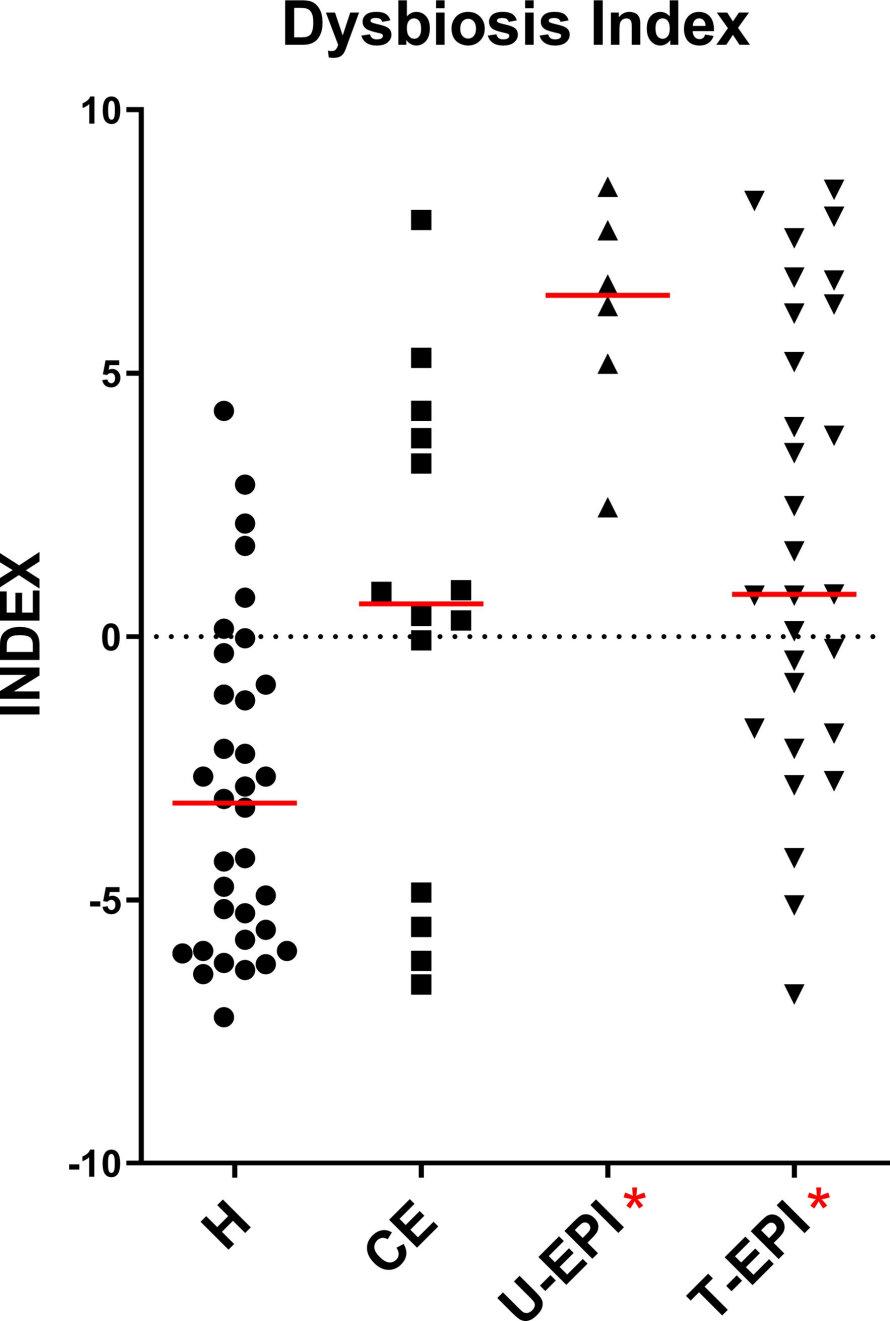
Dysbiosis Index for healthy and diseased dogs. Abbreviations as in Fig 1.

Dogs with untreated and treated EPI had significantly increased D-, L-, and total fecal lactate concentrations (Table 3, Fig 1). One sample from the untreated EPI group had poor quality DNA and therefore was removed from analysis of qPCR and DI. Dogs with untreated and treated EPI had significantly less *Fusobacterium* and *C*. *hiranonis*, and significantly more *E*. *coli*, *Lactobacillus*, and *Bifidobacterium* than healthy control dogs (Table 3, Figs 2 and 4). Only dogs treated for EPI had higher abundance of *Turicibacter*, *Streptococcus*, and *Enterococcus* when compared to healthy control dogs (Table 3, Figs 2 and 4). Dogs untreated for EPI had lower abundance of *Blautia* than healthy control dogs (Table 3, Fig 2). Both untreated and treated dogs with EPI had an increased Dysbiosis Index (Table 3, Fig 5). Both groups of dogs with EPI also had lower concentrations of lithocholic acid, deoxycholic acid, and total secondary bile acids when compared to healthy control dogs (Table 4, Fig 3). Additionally, dogs treated for EPI had higher concentrations of ursodeoxycholic acid than healthy control dogs (Table 4, Fig 3). A subset of dogs treated for EPI (n = 12) had received pancreatic enzyme replacement therapy for 0.08–9.50 years (median 4.45 years); duration was negatively correlated with *Turicibacter* abundance (Spearman’s ρ = -0.634; p = 0.0268).

## Discussion

### Analytical validation of an assay to measure fecal lactate

An enzymatic assay to measure D-, L-, and total lactate in canine feces was successfully established for use in a 96-well plate format. Values obtained for validation variables (S2–S7 Tables) suggest the assay was linear, accurate, precise, and reproducible. Lactate was relatively stable in deproteinized fecal extracts up to 28 days of storage at -80°C. The reference intervals for fecal lactate in healthy dogs (n = 34) were 0.7–1.4 mM, 0.3–6.0 mM, and 1.0–7.0 mM for D-, L-, and total lactate, respectively. The assay had a working range of 0.007 to 0.581 mM for D-lactate, and 0.003 to 0.581 mM for L-lactate, as determined by the lower limit of detection and highest standard concentration. The use of a 96-well plate format allows for more cost- and time-effective measurement of lactate in multiple samples and is achievable in a research setting. For the inter-assay aspect of the validation, 8 aliquots from each of 7 individual homogenized stool samples were used as the replicates, because this would show the summed variation of the extraction procedure plus assay variation. While the variability was moderately high (%CVs of 24%, 20%, and 19% for D-, L-, and total lactate, respectively), there were still large clear differences in fecal lactate concentrations between healthy and diseased populations in this study. This suggests that although the assay may not be useful for detecting small changes in lactate concentrations, it would be useful for clinical samples.

### Quantification of lactate, bile acids, and bacterial groups in canine feces

We quantified D-, L-, and total lactate as well as bile acids and bacterial groups in feces of healthy dogs and dogs with various gastrointestinal diseases. The assay to measure lactate in canine feces has made available the opportunity to obtain concentrations of fecal lactate in studies on gastrointestinal diseases in companion animals. We have directly measured lactate concentrations as well as bacterial abundances of groups thought to produce lactate in the feces of dogs. Significant differences were observed in concentrations of fecal lactate between diseased and healthy dogs (Table 3, Fig 1). Dogs with exocrine pancreatic insufficiency had the highest fecal lactate concentrations, and all diseased groups of dogs had significantly increased concentrations of fecal D- and L-lactate, suggesting that there is some accumulation of luminal lactate in GI diseases.

There are several theories why lactate accumulates in the GI tract. Ewaschuk et al. [30] describes the process in short bowel syndrome as a series of events: poor carbohydrate digestion results in sugars being delivered to the colon, where the pH is decreased by organic acid production through fermentation, finally resulting in acid-resistant *Lactobacillus* spp. growing preferentially.

Interestingly, we found that fecal lactate and LAB were increased in dogs with EPI (Table 3, Figs 1 and 4), a disease also characterized by maldigestion. The taxa *Lactobacillus* spp. and *Bifidobacterium* spp. were increased in both groups of dogs with EPI, while *Enterococcus* was increased only in dogs receiving enzyme replacement therapy for EPI. The small number of animals analyzed in the untreated EPI group (n = 7) is a limitation to this study, and more samples would be needed to accurately determine relative bacterial abundances in untreated dogs. For ethical reasons, it is difficult to obtain fecal samples from dogs diagnosed with EPI that have not received treatment unless recruited at diagnosis. Additionally, this theory of lactate accumulation from bacterial production might not explain lactate accumulation in all cases, as shown by the increased fecal lactate in dogs with chronic enteropathy without concurrent increases in lactate-producing bacterial groups.

*In vitro* studies by Belenguer et al. [47] point to a different reason for lactate accumulation; their work suggests that once the intraluminal pH decreases past a certain point, lactate production will be maintained but lactate utilization will decrease. Future work could examine lactate-consuming bacterial groups or look at the microbial metabolites in a broader sense to understand the metabolic changes occurring in disease states. Our findings suggest that an increase in luminal D-lactate appears to occur frequently in patients with GI disease that did not have neurological signs characteristic of D-lactic acidosis. Previous studies in humans have also described an increase in fecal lactate without concurrent increase in plasma levels, in both patients with short bowel syndrome [18] and healthy individuals fed lactulose [19]. These results and our findings suggest that increased fecal lactate concentrations alone may not pose an acid/base threat, and that altered absorption or metabolism pathways are likely involved together with an altered microbiota to produce D-lactic acidosis.

Dogs with EPI had a significant dysbiosis, as indicated by an increased Dysbiosis Index compared to healthy dogs (Table 3, Fig 5). It is possible that pancreatic enzymes could have an effect on the microbiota independently of the disease, as they have been shown to do in mice [48]. However, the effect of the disease is likely larger than the effect of the treatment since untreated dogs with EPI also had a significant dysbiosis (Table 3, Fig 5). Dogs with CE did not have a significantly increased Dysbiosis Index despite *Faecalibacterium* being decreased, possibly due to a lack of statistical power. However, when proportions of dogs with Dysbiosis Indexes above and below zero were compared, healthy dogs were more likely to have a DI less than zero when compared to dogs with CE (82% versus 36% respectively, p = 0.0044, Fisher’s exact test). Other studies have consistently shown dogs with CE to have an increased DI when compared to healthy dogs [8, 10, 49].

It remains unclear whether microbial dysbiosis is a contributing factor in EPI. Nonetheless, describing alterations in the microbiota is an important step in the development of novel therapeutics, especially for dogs with CE or EPI that do not respond to conventional treatments alone. Recent studies in dogs have supported the idea that altering the microbiota can aid in treatment of diarrheal diseases. Pereira et al. showed that fecal microbiota transplantations in puppies with parvovirus infection were associated with faster resolution of diarrhea [50]. Numerous studies have shown benefits of probiotics in dogs with gastrointestinal diseases [51–55]. However, the literature surrounding dogs with EPI is mostly focused on antibiotic administration, and other potentially more beneficial methods of altering the intestinal microbiota should be explored.

One potential limitation of this study is the lack of sufficient detail in dietary histories to be able to calculate exact macronutrient contents. However, the vast majority of dogs in all groups were being fed commercial maintenance diets that contain similar amounts of macronutrients (i.e., 20–30% protein and 10–20% fat guaranteed analysis; Table 1). Previous studies have shown that large differences in dietary macronutrients can affect the microbiota [56, 57]. Li et al. compared two diets with a 23.9% difference in protein and a 27.9% difference in carbohydrate content and found that these large macronutrient differences affected bacterial abundances within the *Firmicutes* and *Bacteroidetes* phyla, including *C*. *hiranonis*, and was independent of breed [57]. Our study included a variety of dog breeds with a relatively large proportion of German Shepherd Dogs, but a previous study by Isaiah et al. showed that this breed did not differ in their microbial communities compared to other breeds [3].

In our study, one dog in the CE group was being fed a homemade only raw meat based diet, which another study showed decreases the proportion of *Lactobacillus* and increases fecal lactate concentrations compared to commercial diets [58]. However, studies have also shown that the effect of diet or dietary protein source on the microbiota is less than the effect of disease [59, 60]. Additionally, since client-owned dogs are often fed treats and/or table scraps in addition to their regular diet, it is difficult to retrospectively evaluate the effect of diet in this population.

Secondary bile acids were significantly decreased in all diseased groups compared to healthy dogs, and *Clostridium hiranonis* was decreased in dogs with EPI (Tables 3 and 4, Figs 2 and 3). *C*. *hiranonis* is a bacterial group of interest because it has been shown to have high 7α-dehydroxylation activity on primary bile acids [61], suggesting that a decrease in its presence could lead to decreased conversion of primary to secondary bile acids. Our results support this relationship and suggest that this bacterial species may be a large contributor to the secondary bile acid pool in dogs. Although there was not a significant reduction of *C*. *hiranonis* in dogs with CE in this study, others have found decreased *C*. *hiranonis* in dogs with IBD [6] and decreased secondary bile acids in dogs with CE [20, 49]. Additionally, dysbiosis and bile acid dysmetabolism have been shown to occur in humans with IBD [62].

Bile acids have also been implicated in chronic diarrhea in humans [63, 64]. Malabsorption of bile acids leads to a disruption of the enterohepatic loop, and ultimately production and excretion of more primary bile acids into the lumen where they can have secretory effects. Bile acid malabsorption can be measured with serum 7αC4, which is a surrogate marker of hepatic bile acid synthesis [65]. In this study, fecal primary bile acids were not increased in dogs with CE nor dogs with EPI. However, Kent et al. measured serum 7αC4 concentrations in dogs with chronic diarrhea and found increased concentrations in 17.6% of the affected dogs, suggesting bile acid malabsorption may also play a role in some dogs with gastrointestinal disease [66]. Furthermore, a recent study by Giaretta et al. showed that dogs with CE had decreased expression of the apical sodium-dependent bile acid transporter protein in the ileum and increased percentage of primary bile acids in the feces when compared to healthy dogs [49]. In our retrospective analysis for this study, details on portion and extent of the GI tract affected by inflammation were not obtained, however, we can speculate that small intestinal involvement may precede bile acid pool alterations in some dogs.

Our results indicate that bile acid dysmetabolism may play a more significant role in canine GI diseases, since secondary bile acids were disrupted in all diseased groups of dogs. Further studies are necessary to make the functional links between bile acids in the gastrointestinal tract, the microbiota, and gastrointestinal diseases.

## Conclusion

An enzymatic assay to measure D-, L-, and total lactate in canine feces was successfully established for use in a 96-well plate format. The assay was linear, accurate, precise, and reproducible. Significant increases in fecal D- and L-lactate concentrations were observed in dogs with chronic enteropathy and dogs with exocrine pancreatic insufficiency. Although dogs with chronic enteropathy did not have many alterations to individual bacterial groups, they were more likely to have intestinal dysbiosis (DI>0) than healthy control dogs. Dogs with exocrine pancreatic insufficiency had the most profound alterations to their microbiota, as evidenced by their significantly increased Dysbiosis Index and significant increase in lactic acid producing bacteria. Dogs with GI disease also had lower concentrations of secondary bile acids than healthy dogs.

Further studies are necessary to determine the clinical utility of lactate and bile acid quantification in canine feces. Although these metabolites alone may not be good indicators of dysbiosis, when interpreted alongside bacterial abundances, they become promising targets for further elucidating the role of the microbiota in health and disease.

## Supporting information

S1 Formula Calculator A template for calculating final lactate concentrations with adjustments based on starting weight of feces, dilution factor, and dry matter content. (XLSX)

S1 Protocol Quantification of fecal lactate. (DOCX)

S2 Protocol Validation of the assay for measurement of fecal lactate. (DOCX)

S1 Table Primers and cycling conditions used in qPCRs. (PDF)

S2 Table Dilutional parallelism of seven fecal samples. Observed-to-expected ratios are in bold and the minimum, maximum, mean, and standard deviation of those observed-to-expected ratios are provided in the box at the end of the table.
(PDF)

S3 Table Spiking recovery of four canine fecal samples. Observed-to-expected ratios are in bold and the minimum, maximum, mean, and standard deviation of those observed-to-expected ratios are provided in the box at the end of the table.
(PDF)

S4 Table Intra-assay variability of four canine fecal samples. Coefficients of variation are in bold and the minimum, maximum, mean, and standard deviation of these coefficients of variation are provided in the box at the end of the table.
(PDF)

S5 Table Inter-assay variability of seven canine fecal samples. Coefficients of variation are in bold and the minimum, maximum, mean, and standard deviation of these coefficients of variation are provided in the box at the end of the table.
(PDF)

S6 Table Stability of deproteinized fecal extracts for seven canine fecal samples at 4°C for 24 hours. Coefficients of variation are in bold and the mean of these coefficients of variation for D-, L-, and total lactate are provided.
(PDF)

S7 Table Stability of deproteinized fecal extracts for seven canine fecal samples at -80°C for 4 weeks. Coefficients of variation are in bold and the mean of these coefficients of variation for D-, L-, and total lactate are provided.
(PDF)
