# Supplementary material for: Altered microbiota, fecal lactate, and fecal bile acids in dogs with gastrointestinal disease
Source: PLoS One. 2019 Oct 31;14(10):e0224454. doi: 10.1371/journal.pone.0224454 (PMC6822739; doi:10.1371/journal.pone.0224454)
Supplement: S1 Protocol — (DOCX) [file pone.0224454.s002.docx]

# S1 Protocol. Quantification of fecal lactate

## Deproteinization of Feces

Frozen samples were thawed at room temperature for aliquoting. An aliquot of 125 ± 5 mg feces was made for each fecal sample and placed into 1.5 mL microcentrifuge tubes. Aliquots were then stored at -80°C until deproteinization. Deproteinization of fecal samples was achieved by using the protocol of Rul et al. [1] with modifications (<http://dx.doi.org/10.17504/protocols.io.hrqb55w>. [PROTOCOL DOI]). Briefly, 750 µL of 0.1 M triethanolamine buffer (pH 9.15) was added to each fecal aliquot. The tubes were vortexed and placed in the refrigerator (4°C) for three hours, vortexing every hour to ensure thorough mixing. The samples were then centrifuged at 13,000 x g for 5 minutes at 4°C. Next, 495 µL of the supernatant was carefully pipetted off into a new 1.5 mL microcentrifuge tube. These supernate aliquots were stored at -80°C overnight to provide ample time for the next processing steps. From empirical experience, the extra freeze-thaw cycles favorably increased protein pellet size while not affecting lactate concentrations. The next morning, samples were thawed at room temperature for 20-40 minutes. Once thawed, 10 µL of 6 M trichloroacetic acid was added to each sample. The samples were vortexed for 10 seconds and placed in an ice bath for 20 minutes. Then samples were vortexed for a few seconds and centrifuged at 4,500 x g for 20 minutes at 4°C. After centrifugation, a protein pellet was noted in the bottom of each tube. Next, 400 µL of supernatant was pipetted off into a 2 mL microcentrifuge tube and 1,600 µL 0.1 M triethanolamine buffer (pH 9.15) added to achieve a neutral or alkaline pH (between 7 and 10). These deproteinized fecal extracts were either used immediately for lactate analysis or stored in -80°C for later use.

## Spectrophotometric Analysis

Ultraviolet spectrophotometric analysis of fecal lactate was performed using a commercially available enzymatic kit (D-/L-Lactate Enzymatic Kit, R-Biopharm Inc.) with modifications to the manufacturer protocol for use with a 96-well plate format. The concentrations of D- and L-lactate were determined by measuring the sequential formation of NADH by the increase in absorption at 340 nm wavelength following addition of stereospecific D- and L-LDH (Supplemental material; manufacturer protocol for D-/L-lactic acid kit). The protocol was modified for use with a 96-well plate format by dividing all volumes by a factor of ten. Standard dilutions were made for D- and L- lactate by the addition of ultra-pure water (PURELAB® Ultra Water Purification System, ELGA LabWater; Table A). Master mix (MM) solution was made on an as needed basis depending on the number of samples and consisted of solution 1 (100 µL for each sample), 2 (20 µL for each sample), and 3 (2 µL for each sample) mixed in a 15 or 50 mL tube (Table B). Blanks, standards, and samples were pipetted in duplicate onto a 96-well plate (Table C) and placed on a plate shaker for one minute. After a 15-minute incubation at 25°C, the first absorbance (A1) was read at 340 nm wavelength. Then 2 µL D-LDH was added to each well, shook for one minute, and incubated at 25°C for 30 minutes. The second absorbance (A2) reading was taken and then 2 µL L-LDH added to each well, shook and incubated at 25°C for 30 minutes. The third absorbance (A3) reading was taken and then data processing was performed. A path length adjustment setting was implemented on the plate reader software (Gen5 v. 2.07, BioTek® Instruments, Inc.) to account for using a protocol originally designed for use with 1 cm diameter cuvettes. Standard curve and lactate concentrations were calculated. The standard curve was set to be quadratic and lactate concentrations were adjusted by subtracting the absorbance difference of the blank. Then, if D- or L-lactate concentrations were below their respective lower limits of quantification (g/L), concentrations were adjusted to 0.002 g/L for D-lactate or 0.0007 g/L for L-lactate. Final lactate concentrations were also adjusted based on starting weight of the feces, dilution factor, and dry matter content (S1 Formula Calculator).

**Table A.** Standard dilutions of D- and L-lactate.

| **Standard** | **Dilution** | **Concentration (g/L)** |
| --- | --- | --- |
| 1 | 1:4 | 0.05175 |
| 2 | 1:10 | 0.0207 |
| 3 | 1:20 | 0.01035 |
| 4 | 1:40 | 0.005175 |
| 5 | 1:80 | 0.0025875 |
| Stock concentration = 0.207 g/L | | |

**Table B.** Description of solutions included in enzymatic kit.

| **Solution Number** | **Description*** |
| --- | --- |
| 1 | approx. 30 ml solution, consisting of: glycylglycine buffer, pH approx. 10.0; L-glutamic acid, approx. 440 mg |
| 2 | approx. 210 mg NAD, lyophilizate, reconstituted in 6 ml redist. Water |
| 3 | approx. 0.7 ml glutamate-pyruvate transaminase suspension, approx. 1100 U |
| 4 | approx. 0.7 ml D-lactate dehydrogenase solution, approx. 3800 U |
| 5 | approx. 0.7 ml L-lactate dehydrogenase solution, approx. 3800 U |
| *Descriptions obtained from manufacturer protocol | |

**Table C.** Samples and reagent volumes.

| **Well type** | **Volumes** |
| --- | --- |
| blanks | 122 µL MM, 100 µL water |
| standards (D- and L-lactate) | 122 µL MM, 100 µL standards 1-5 |
| unknown samples | 122 µL MM, 100 µL fecal extract |
| MM = master mix | |

## References

1. Rul F, Ben-Yahia L, Chegdani F, Wrzosek L, Thomas S, Noordine M-L, et al. Impact of the metabolic activity of Streptococcus thermophilus on the colon epithelium of gnotobiotic rats. J Biol Chem. 2011;286(12):10288-96. doi: 10.1074/jbc.M110.168666. PubMed Central PMCID: PMCPMC3060483.
