# Supplementary material for: Altered microbiota, fecal lactate, and fecal bile acids in dogs with gastrointestinal disease
Source: PLoS One. 2019 Oct 31;14(10):e0224454. doi: 10.1371/journal.pone.0224454 (PMC6822739; doi:10.1371/journal.pone.0224454)
Supplement: S2 Protocol — (DOCX) [file pone.0224454.s003.docx]

# S2 Protocol. Validation of the assay for measurement of fecal lactate

Lower limit of detection (LLOD) and lower limit of quantification (LLOQ) were assessed by measuring ten duplicates of the blank and calculating the mean and standard deviation of the absorbance differences, A2-A1 (D-lactate) and A3-A2 (L-lactate). The analytical sensitivity (S) at the lower end of the standard curve was calculated by:

$$S=\frac{\Delta concentration}{\Delta intensity}$$

where Δ concentration = (concentration standard 4) – (concentration standard 5) = 0.00259 g/L, and Δ intensity = (ΔA standard 4- ΔA standard 5)-ΔA_blank_. (ΔA standards calculated from average of 8 runs). Next LLOD and LLOQ were calculated by the following equations:

LLOD = ks_bl_S

LLOQ = ks_bl_S

where k is chosen based on desired level of confidence (k=3 for LLOD and k=10 for LLOQ) and s_bl_ is the standard deviation of the blank.

Assay linearity was evaluated by assessing dilutional parallelism for seven different fecal samples at dilutions of 1, 1:2, 1:4, 1:10, 1:20, 1:40, and 1:80 for each sample. Dilutions were performed by the addition of ultra-pure water (PURELAB® Ultra Water Purification System, ELGA LabWater). The accuracy of the assay was measured by mixing previously quantified extracts of four samples in a 1:1 ratio. The percentage of lactate recovery was calculated as the observed-to-expected ratio (OE%):

$$OE\%=\left[ \frac{observed value \left( \mathrm{mM} \right)}{expected value \left( \mathrm{mM} \right)} \right]\times100$$

To evaluate precision of the assay, four different fecal samples that spanned the working range of the assay were analyzed 8 times within the same assay run on one single plate. The intra-assay coefficient of variation (%CV) was calculated as:

$$\%CV=\left( \frac{standard deviation}{\mathrm{mean}} \right)\times100$$

The reproducibility of the assay was evaluated by analyzing seven different fecal samples in 8 separate assay runs on different days, followed by calculation of inter-assay %CVs. All fecal validation samples were run in duplicate and at dilutions that allowed them to fall within the working range of the assay.
