# Supplementary material for: Altered microbiota, fecal lactate, and fecal bile acids in dogs with gastrointestinal disease
Source: PLoS One. 2019 Oct 31;14(10):e0224454. doi: 10.1371/journal.pone.0224454 (PMC6822739; doi:10.1371/journal.pone.0224454)
Supplement: S1 Table — (PDF) [file pone.0224454.s004.pdf]

S1 Table. Primers and cycling conditions used in qPCRs.

Target	Primer sequences (5' - 3')	Initial denaturing temp(°C), time	# of cycles	Denaturing temp(°C), time	Annealing temp(°C), time	Reference
Universal	F-CCTACGGGAGGCAGCAGT<br>R-ATTACCGCGGCTGCTGG	98,<br>2 min	35	98,<br>5 sec	59,<br>5 sec	F primer [1];<br>R primer [2]
Faecalibacterium spp.	F-GAAGGCGGCCTACTGGGCAC<br>R-GTGCAGGCGAGTTGCAGCCT	98,<br>2 min	40	98,<br>5 sec	60,<br>5 sec	[3]
Turicibacter spp.	F-CAGACGGGGACAACGATTGGA<br>R-TACGCATCGTCGCCTTGGTA	98,<br>2 min	40	98,<br>3 sec	57,<br>3 sec	[4]
Streptococcus spp.	F-TTATTTGAAAGGGGCAATTGCT<br>R-GTGAACTTTCCACTCTCACAC	95,<br>2 min	40	95,<br>5 sec	54,<br>10 sec	[5]
Escherichia coli	F-GTTAATACCTTTGCTCATTGA<br>R-ACCAGGGTATCTAATCCTGTT	98,<br>2 min	40	98,<br>3 sec	55,<br>3 sec	[6]
Blautia spp.	F-TCTGATGTGAAAGGCTGGGGCTTA<br>R-GGCTTAGCCACCCGACACCTA	98,<br>2 min	40	98,<br>4 sec	56,<br>4 sec	[4]
Fusobacterium spp.	F-KGGGCTCAACMCMGTATTGCGT<br>R-TCGCGTTAGCTTGGGCGCTG	98,<br>2 min	40	98,<br>4 sec	50.5,<br>4 sec	[4]
Clostridium hiranonis	F-AGTAAGCTCCTGATACTGTCT<br>R-AGGGAAAGAGGAGATTAGTCC	95,<br>3 min	40	95,<br>30 sec	59,<br>5 sec	[7]
Lactobacillus spp.	F-AGCAGTAGGGAATCTTCCA*<br>R-CACCGCTACACATGGAG**	95,<br>2 min	40	95,<br>5 sec	58,<br>10 sec	[8]
Bifidobacterium spp.	F-TCGCGTCYGGTGTGAAAG<br>R-CCACATCCAGCRTCCAC	98,<br>2 min	40	98,<br>3 sec	60,<br>3 sec	[9]
Enterococcus spp.	F-CCCTTATTGTTAGTTGCCATCATT<br>R-ACTCGTTGTACTTCCCATTGT	98,<br>3 min	40	98,<br>3 sec	61,<br>3 sec	[9]

*Originally described by Walter et al., 2001 [10].
**Originally described by Heilig et al., 2002 [11].

References

1. Nadkarni MA, Martin FE, Jacques NA, Hunter N. Determination of bacterial load by real-time PCR using a broad-range (universal) probe and primers set. Microbiology (Reading, England). 2002;148(1):257-66. doi: 10.1099/00221287-148-1-257.
2. Muyzer G, de Waal EC, Uitterlinden AG. Profiling of complex microbial populations by denaturing gradient gel electrophoresis analysis of polymerase chain reaction-amplified genes coding for 16S rRNA. Applied and Environmental Microbiology. 1993;59(3):695-700.
3. Garcia-Mazcorro JF, Suchodolski JS, Jones KR, Clark-Price SC, Dowd SE, Minamoto Y, et al. Effect of the proton pump inhibitor omeprazole on the gastrointestinal bacterial microbiota of healthy dogs. FEMS Microbiol Ecol. 2012;80(3):624-36. doi: 10.1111/j.1574-6941.2012.01331.x.
4. Suchodolski JS, Markel ME, Garcia-Mazcorro JF, Unterer S, Heilmann RM, Dowd SE, et al. The fecal microbiome in dogs with acute diarrhea and idiopathic inflammatory bowel disease. PLoS One. 2012;7(12):e51907. doi: 10.1371/journal.pone.0051907.
5. Furet JP, Quenee P, Tailliez P. Molecular quantification of lactic acid bacteria in fermented milk products using real-time quantitative PCR. Int J Food Microbiol. 2004;97(2):197-207. doi: 10.1016/j.ijfoodmicro.2004.04.020.
6. Malinen E, Kassinen A, Rinttila T, Palva A. Comparison of real-time PCR with SYBR Green I or 5'-nuclease assays and dot-blot hybridization with rDNA-targeted oligonucleotide probes in quantification of selected faecal bacteria. Microbiology (Reading, England). 2003;149(Pt 1):269-77. doi: 10.1099/mic.0.25975-0.
7. Kitahara M, Sakamoto M, Benno Y. PCR detection method of Clostridium scindens and C. hiranonis in human fecal samples. Microbiol Immunol. 2001;45(3):263-6.
8. Malinen E, Rinttila T, Kajander K, Matto J, Kassinen A, Krogius L, et al. Analysis of the fecal microbiota of irritable bowel syndrome patients and healthy controls with real-time PCR. The American journal of gastroenterology. 2005;100(2):373-82. doi: 10.1111/j.1572-0241.2005.40312.x.
9. Rinttila T, Kassinen A, Malinen E, Krogius L, Palva A. Development of an extensive set of 16S rDNA-targeted primers for quantification of pathogenic and indigenous bacteria in faecal samples by real-time PCR. J Appl Microbiol. 2004;97(6):1166-77. doi: 10.1111/j.1365-2672.2004.02409.x.
10. Walter J, Hertel C, Tannock GW, Lis CM, Munro K, Hammes WP. Detection of Lactobacillus, Pediococcus, Leuconostoc, and Weissella species in human feces by using group-specific PCR primers and denaturing gradient gel electrophoresis. Appl Environ Microbiol. 2001;67(6):2578-85. doi: 10.1128/aem.67.6.2578-2585.2001.
11. Heilig HG, Zoetendal EG, Vaughan EE, Marteau P, Akkermans AD, de Vos WM. Molecular diversity of Lactobacillus spp. and other lactic acid bacteria in the human intestine as determined by specific amplification of 16S ribosomal DNA. Appl Environ Microbiol. 2002;68(1):114-23.
