# Supplementary material for: Altered microbiota, fecal lactate, and fecal bile acids in dogs with gastrointestinal disease
Source: PLoS One. 2019 Oct 31;14(10):e0224454. doi: 10.1371/journal.pone.0224454 (PMC6822739; doi:10.1371/journal.pone.0224454)
Supplement: S2 Table — Observed-to-expected ratios are in bold and the minimum, maximum, mean, and standard deviation of those observed-to-expected ratios are provided in the box at the end of the table. (PDF) [file pone.0224454.s005.pdf]

S2 Table. Dilutional parallelism of seven fecal samples. Observed-to-expected ratios are in bold and the minimum, maximum, mean, and standard deviation of those observed-to-expected ratios are provided in the box at the end of the table.

	D-lactate			L-lactate			total lactate		
**Sample 1**	O	E	**OE%**	O	E	**OE%**	O	E	**OE%**
S1	HIGH			HIGH					
S1 1:2	HIGH	N/A	N/A	HIGH	N/A	N/A	N/A	N/A	N/A
S1 1:4	0.047	N/A	N/A	HIGH	N/A	N/A	N/A	N/A	N/A
S1 1:10	0.021	0.019	**112**	HIGH	N/A	N/A	N/A	N/A	N/A
S1 1:20	0.011	0.009	**112**	0.033	N/A	N/A	0.043	N/A	N/A
S1 1:40	0.005	0.005	**102**	0.014	0.016	**87**	0.019	0.022	**88**
S1 1:80	LOW	0.002	N/A	0.007	0.008	**91**	N/A	N/A	N/A
**Sample 2**									
S2	HIGH			HIGH					
S2 1:2	HIGH	N/A	N/A	HIGH	N/A	N/A	N/A	N/A	N/A
S2 1:4	0.030	N/A	N/A	HIGH	N/A	N/A	N/A	N/A	N/A
S2 1:10	0.013	0.012	**106**	HIGH	N/A	N/A	N/A	N/A	N/A
S2 1:20	0.005	0.006	**89**	0.028	N/A	N/A	0.033	N/A	N/A
S2 1:40	0.003	0.003	**92**	0.014	0.014	**104**	0.017	0.017	**104**
S2 1:80	LOW	0.002	N/A	0.007	0.007	**100**	N/A	N/A	N/A
**Sample 3**									
S3	HIGH			HIGH					
S3 1:2	0.033	N/A	N/A	HIGH	N/A	N/A	N/A	N/A	N/A
S3 1:4	0.017	0.016	**103**	HIGH	N/A	N/A	N/A	N/A	N/A
S3 1:10	0.007	0.007	**107**	0.032	N/A	N/A	0.039	N/A	N/A
S3 1:20	0.003	0.003	**93**	0.017	0.016	**105**	0.020	0.020	**102**
S3 1:40	LOW	0.002	N/A	0.009	0.008	**109**	N/A	N/A	N/A
S3 1:80	LOW	0.001	N/A	0.005	0.004	**112**	N/A	N/A	N/A
**Sample 4**									
S4	HIGH			HIGH					
S4 1:2	0.048	N/A	N/A	HIGH	N/A	N/A	N/A	N/A	N/A
S4 1:4	0.028	0.024	**117**	HIGH	N/A	N/A	N/A	N/A	N/A
S4 1:10	0.011	0.010	**117**	0.028	N/A	N/A	0.039	N/A	N/A
S4 1:20	0.005	0.005	**99**	0.013	0.014	**96**	0.018	0.019	**93**
S4 1:40	LOW	0.002	N/A	0.007	0.007	**102**	N/A	N/A	N/A
S4 1:80	LOW	0.001	N/A	0.003	0.003	**101**	N/A	N/A	N/A

S2 Table Continued

	D-lactate			L-lactate			total lactate		
**Sample 5**	O	E	**OE%**	O	E	**OE%**	O	E	**OE%**
S5	0.044			HIGH					
S5 1:2	0.020	0.022	**89**	0.044	N/A	N/A	0.064	N/A	N/A
S5 1:4	0.011	0.011	**96**	0.020	0.022	**91**	0.031	0.032	**96**
S5 1:10	LOW	0.004	N/A	0.008	0.009	**89**	N/A	N/A	N/A
S5 1:20	LOW	0.002	N/A	0.004	0.004	**96**	N/A	N/A	N/A
S5 1:40	LOW	0.001	N/A	LOW	0.002	N/A	N/A	N/A	N/A
S5 1:80	LOW	0.001	N/A	LOW	0.001	N/A	N/A	N/A	N/A
**Sample 6**									
S6	0.007			0.027			0.034		
S6 1:2	0.003	0.004	**93**	0.014	0.013	**102**	0.017	0.017	**101**
S6 1:4	LOW	0.002	N/A	0.007	0.007	**111**	N/A	N/A	N/A
S6 1:10	LOW	0.001	N/A	0.003	0.003	**109**	N/A	N/A	N/A
S6 1:20	LOW	0.000	N/A	LOW	0.001	N/A	N/A	N/A	N/A
S6 1:40	LOW	0.000	N/A	LOW	0.001	N/A	N/A	N/A	N/A
S6 1:80	LOW	0.000	N/A	LOW	0.000	N/A	N/A	N/A	N/A
**Sample 7**									
S7	0.011			0.020			0.031		
S7 1:2	0.005	0.005	**92**	0.010	0.010	**101**	0.015	0.015	**98**
S7 1:4	LOW	0.003	N/A	0.005	0.005	**100**	N/A	N/A	N/A
S7 1:10	LOW	0.001	N/A	LOW	0.002	N/A	N/A	N/A	N/A
S7 1:20	LOW	0.001	N/A	LOW	0.001	N/A	N/A	N/A	N/A
S7 1:40	LOW	0.000	N/A	LOW	0.000	N/A	N/A	N/A	N/A
S7 1:80	LOW	0.000	N/A	LOW	0.000	N/A	N/A	N/A	N/A

	**D-lactate**	**L-lactate**	**total lactate**
**min**	92	89	88
**max**	111	109	104
**mean**	99	100	97
**SD**	7	7	5

O = observed lactate concentration (g/L), E = expected lactate concentration (g/L), OE% = observed-to-expected ratio.
