# Supplementary material for: Altered microbiota, fecal lactate, and fecal bile acids in dogs with gastrointestinal disease
Source: PLoS One. 2019 Oct 31;14(10):e0224454. doi: 10.1371/journal.pone.0224454 (PMC6822739; doi:10.1371/journal.pone.0224454)
Supplement: S3 Table — Observed-to-expected ratios are in bold and the minimum, maximum, mean, and standard deviation of those observed-to-expected ratios are provided in the box at the end of the table. (PDF) [file pone.0224454.s006.pdf]

S3 Table. Spiking recovery of four canine fecal samples. Observed-to-expected ratios are in bold and the minimum, maximum, mean, and standard deviation of those observed-to-expected ratios are provided in the box at the end of the table.

	D-lactate (mM)			L-lactate (mM)			total lactate (mM)		
**Sample A**	102			237			339		
**Sample B**	27			66			92		
**Sample C**	13			27			40		
**Sample C**	4			6			10		
	D-lactate			L-lactate			total lactate		
	O	E	**OE%**	O	E	**OE%**	O	E	**OE%**
A+B	62	64	**96**	155	151	**102**	216	215	**100**
A+C	58	58	**101**	127	132	**96**	185	189	**98**
A+D	53	53	**100**	121	122	**100**	174	174	**100**
B+A	62	64	**96**	155	151	**102**	216	215	**100**
B+C	20	20	**100**	55	46	**119**	75	66	**113**
B+D	15	15	**98**	40	36	**113**	55	51	**108**
C+A	58	58	**101**	127	132	**96**	185	189	**98**
C+B	20	20	**100**	55	46	**119**	75	66	**113**
C+D	9	9	**103**	17	16	**100**	25	25	**101**
D+A	53	53	**100**	121	122	**100**	174	174	**100**
D+B	15	15	**98**	40	36	**113**	55	51	**108**
D+C	9	9	**103**	17	16	**100**	25	25	**101**
	**D-lactate**			**L-lactate**			**total lactate**		
**min**	96			96			98		
**max**	103			119			113		
**mean**	100			105			103		
**SD**	2			8			5		

O = observed lactate concentration (mM), E = expected lactate concentration (mM), OE% = observed-to-expected ratio, SD = standard deviation.
