# Supplementary material for: Altered microbiota, fecal lactate, and fecal bile acids in dogs with gastrointestinal disease
Source: PLoS One. 2019 Oct 31;14(10):e0224454. doi: 10.1371/journal.pone.0224454 (PMC6822739; doi:10.1371/journal.pone.0224454)
Supplement: S4 Table — Coefficients of variation are in bold and the minimum, maximum, mean, and standard deviation of these coefficients of variation are provided in the box at the end of the table. (PDF) [file pone.0224454.s007.pdf]

S4 Table. Intra-assay variability of four canine fecal samples. Coefficients of variation are in bold and the minimum, maximum, mean, and standard deviation of these coefficients of variation are provided in the box at the end of the table.

sample	1	2	3	4
number of repeats	8	8	8	8
	**D-lactate**			
mean (mM)	109	22	14	6
standard deviation (mM)	6	1	1	0
**% CV**	**6**	**4**	**7**	**4**
	**L-lactate**			
mean (mM)	217	123	25	10
standard deviation (mM)	16	7	1	0
**% CV**	**7**	**6**	**4**	**3**
	**total lactate**			
mean (mM)	327	145	39	16
standard deviation (mM)	19	7	1	0
**% CV**	**6**	**5**	**3**	**3**

	**D-lactate**	**L-lactate**	**total lactate**
**min**	4	3	3
**max**	7	7	6
**mean**	5	5	4
**SD**	1	2	1

%CV = coefficient of variation, SD = standard deviation.
