# Supplementary material for: Altered microbiota, fecal lactate, and fecal bile acids in dogs with gastrointestinal disease
Source: PLoS One. 2019 Oct 31;14(10):e0224454. doi: 10.1371/journal.pone.0224454 (PMC6822739; doi:10.1371/journal.pone.0224454)
Supplement: S5 Table — Coefficients of variation are in bold and the minimum, maximum, mean, and standard deviation of these coefficients of variation are provided in the box at the end of the table. (PDF) [file pone.0224454.s008.pdf]

S5 Table. Inter-assay variability of seven canine fecal samples. Coefficients of variation are in bold and the minimum, maximum, mean, and standard deviation of these coefficients of variation are provided in the box at the end of the table.

sample	1	2	3	4	5	6	7
number of repeats	8	7*	8	8	8	8	8
	**D-lactate**						
mean (mM)	75	106	20	29	15	3	4
standard deviation (mM)	4	6	7	8	1	2	1
**% CV**	**6**	**5**	**32**	**29**	**5**	**56**	**31**
	**L-lactate**						
mean (mM)	233	224	143	76	30	7	7
standard deviation (mM)	12	21	19	15	3	4	2
**% CV**	**5**	**10**	**13**	**20**	**11**	**55**	**28**
	**total lactate**						
mean (mM)	309	331	163	105	45	10	12
standard deviation (mM)	16	24	18	23	4	5	3
**% CV**	**5**	**7**	**11**	**22**	**8**	**54**	**28**

	**D-lactate**	**L-lactate**	**total lactate**
**min**	5	5	5
**max**	56	55	54
**mean**	23	20	19
**SD**	18	16	16

*one repeat was thrown out due to pipetting error
%CV = coefficient of variation, SD = standard deviation.
