# Supplementary material for: Altered microbiota, fecal lactate, and fecal bile acids in dogs with gastrointestinal disease
Source: PLoS One. 2019 Oct 31;14(10):e0224454. doi: 10.1371/journal.pone.0224454 (PMC6822739; doi:10.1371/journal.pone.0224454)
Supplement: S6 Table — Coefficients of variation are in bold and the mean of these coefficients of variation for D-, L-, and total lactate are provided. (PDF) [file pone.0224454.s009.pdf]

S6 Table. Stability of deproteinized fecal extracts for seven canine fecal samples at 4°C for 24 hours. Coefficients of variation are in bold and the mean of these coefficients of variation for D-, L-, and total lactate are provided.

**D-lactate**					
sample	inter-assay mean (mM)	initial (mM)	24h fridge (mM)	% change	**%CV**
1	75	81	81	-2	**0**
2	106	117	100	5	**8**
3	20	10	15	0	**22**
4	29	12	18	6	**19**
5	15	16	14	6	**9**
6	3	3	3	-1	**1**
7	4	3	3	7	**0**
				**Mean %CV = 9**	
**L-lactate**					
1	233	252	240	1	**2**
2	224	242	239	-1	**1**
3	143	160	167	-5	**2**
4	76	46	43	14	**3**
5	30	34	29	4	**8**
6	7	8	9	-10	**7**
7	7	7	6	9	**7**
				**Mean %CV = 4**	
**total lactate**					
1	309	333	321	0	**2**
2	331	360	339	1	**3**
3	163	169	182	-5	**3**
4	105	58	61	12	**3**
5	45	50	43	5	**8**
6	10	11	12	-8	**5**
7	12	10	9	8	**5**
				**Mean %CV = 4**	

%CV = coefficient of variation.
