# Supplementary material for: Altered microbiota, fecal lactate, and fecal bile acids in dogs with gastrointestinal disease
Source: PLoS One. 2019 Oct 31;14(10):e0224454. doi: 10.1371/journal.pone.0224454 (PMC6822739; doi:10.1371/journal.pone.0224454)
Supplement: S7 Table — Coefficients of variation are in bold and the mean of these coefficients of variation for D-, L-, and total lactate are provided. (PDF) [file pone.0224454.s010.pdf]

S7 Table. Stability of deproteinized fecal extracts for seven canine fecal samples at -80°C for 4 weeks. Coefficients of variation are in bold and the mean of these coefficients of variation for D-, L-, and total lactate are provided.

sample	inter-assay mean (mM)	initial (mM)	4wk freezer (mM)	% change	**%CV**
**D-lactate**					
1	75	71	72	1	**1**
2	106	102	114	-5	**5**
3	20	16	14	12	**8**
4	29	24	25	2	**3**
5	15	15	18	-10	**9**
6	3	4	4	-8	**2**
7	4	3	2	13	**1**
				**Mean %CV = 4**	
**L-lactate**					
1	233	219	182	10	**9**
2	224	185	191	3	**2**
3	143	120	122	4	**1**
4	76	64	66	3	**2**
5	30	23	21	10	**4**
6	7	10	9	-1	**7**
7	7	5	4	13	**2**
				**Mean %CV = 4**	
**total lactate**					
1	309	290	253	8	**7**
2	331	287	305	1	**3**
3	163	136	136	5	**0**
4	105	87	91	3	**2**
5	45	38	39	3	**1**
6	10	14	13	-3	**5**
7	12	7	7	13	**2**
				**Mean %CV = 3**	

%CV = coefficient of variation
